# Association between High-Sensitivity C-Reactive Protein and Blood Pressure among Children with History of Low Birth Weight Appropriate for Gestational Age, Low Birth Weight Small for Gestational Age, and Normal Birth Weight in Manado, North Sulawesi

**DOI:** 10.1155/2019/3263264

**Published:** 2019-12-14

**Authors:** Adrian Umboh, Rocky Wilar, Valentine Umboh, Adi Suryadinata Krisetya

**Affiliations:** ^1^Pediatric Nephrology Division, Department of Pediatrics, Faculty of Medicine, Sam Ratulangi University, Manado, Indonesia; ^2^Neonatology Division, Department of Pediatrics, Faculty of Medicine, Prof. Dr. R. D. Kandou General Hospital, Manado, Indonesia; ^3^Department of Pediatrics, Faculty of Medicine, Prof. Dr. R. D. Kandou General Hospital, Manado, Indonesia

## Abstract

Over the past years, low birth weight (LBW) has been proven to be attributed to a wide variety of long-term morbidities, including hypertension. This study aimed to investigate the association between high-sensitivity C-reactive protein (hs-CRP) and blood pressure (BP) in children with a history of LBW appropriate for gestational age (LBW AGA), LBW small for gestational age (LBW SGA), and normal birth weight appropriate for gestational age (NBW AGA). The study cohort comprised children aged 9–12 years who were born in 2007–2010 at Prof. Dr. R. D. Kandou General Hospital Manado and resided in the city of Manado from March to August 2019. The children who met the inclusion criteria were evaluated for BP and hs-CRP level. A total of 120 children who met the inclusion criteria were enrolled in this study. Analysis for the association between LBW and NBW with systolic blood pressure (SBP) showed statistical significance (*p*=0.007). Linear regression analysis indicated a strongly significant influence of BW on serum hs-CRP level and SBP. Every 1 g increase in BW results in a decrease of serum hs-CRP level of 0.001 mg/L. Every 1 g increase in BW is attributed to 0.004 mmHg decrease in SBP. An increase in hs-CRP by 1 mg/L increases the SBP by 4.99 mmHg and DBP by 2.88 mmHg. LBW significantly correlates with hs-CRP level and higher SBP. A comprehensive education must be undertaken for the families who have children with LBW to reduce the risk of developing hypertension later in their life.

## 1. Introduction

Low birth weight (LBW) newborns represent both term babies who experienced intrauterine growth restriction (IUGR) and preterm babies with and without IUGR [1]. Until recently, LBW is still considered as an important public health issue owing to the short- and the long-term consequences it possesses [2, 3]. The prevalence of LBW in Indonesia is around 7–17%. In 2003, the incidence of LBW at Prof. Dr. R. D. Kandou General Hospital Manado was reported to be 10.83%, where 37.70% of the incidence was IUGR [4].

Over the past years, LBW has been proven to be attributed to a wide variety of long-term morbidities, including hypertension, end-stage renal disease, insulin resistance, and cardiovascular diseases [5, 6]. Inflammation is involved in the pathophysiology of cardiovascular diseases. High-sensitivity C-reactive protein (hs-CRP) is one of the most important inflammatory markers. An increase in serum CRP level is associated with atherosclerotic plaque formation. However, current conventional CRP level measurement methods are not sensitive enough to evaluate a very mild increase in serum CRP level [7].

Studies addressing hs-CRP and its role in cardiovascular diseases have been performed in adult patients. However, such studies conducted in pediatric population are still limited, particularly in children with a history of low birth weight. Hence, a study investigating the association between hs-CRP and blood pressure in children with a history of low birth weight appropriate for gestational age (LBW AGA), low birth weight small for gestational age (LBW SGA), and normal birth weight appropriate for gestational age (NBW AGA) is necessary.

## 2. Material and Methods

### 2.1. Study Design

This study is an observational study performed using cross-sectional design. This study was designed to answer a hypothetical theory which states that there is significant correlation between hs-CRP and blood pressure in children with a history of low birth weight.

### 2.2. Ethical Approval

The study protocol was reviewed and approved by the Research Ethical Committee and Health Development of Prof. Dr. R. D. Kandou General Hospital with the reference number 228/EC-KEPK/XI/2018.

### 2.3. Study Setting

The study involved children born at Prof. Dr. R. D. Kandou General Hospital Manado from 2007 to 2010 and resided in the city of Manado from March–August 2019. Prof. Dr. R. D. Kandou is a Teaching Hospital as well as the National Referral Center for the eastern region of Indonesia.

### 2.4. Recruitment and Enrollment

The study cohort comprised children aged 9–12 years who were born in 2007–2010 at Prof. Dr. R. D. Kandou General Hospital Manado and resided in the city of Manado from March–August 2019. The inclusion criteria were children with a history of low (<2500 grams) and normal (2500–4000 grams) birth weight, complete medical record, presented with normal clinical and nutritional status, and whose parents agreed to participate and provided their written informed consent for participation. The exclusion criteria consisted of babies with multiple congenital anomalies, diabetes mellitus, history of medical therapy within the last 2 weeks, and history of renal disease (nephrotic syndrome, acute/chronic glomerulonephritis, and acute/chronic renal failure). To estimate the sample size, the correlative sample size formula was used and a minimum of 97 samples suffice to generate statistical significance.

Sample-size estimation for correlation coefficients [8]:

(1) $$\begin{array}{c} n=\left[\frac{{\left(Z_{1-\alpha }+Z_{1-\beta }\right)}^{2}}{{\left(\left(1/2\right)\ln\left(\left(1+\rho_{0}\right)/\left(1-\rho_{0}\right)\right)\right)}^{2}}\right]+3, \end{array}$$

where *n* is the estimated size of the study sample; *Z*_1−*α*_ is the value in table *Z* (if *α* (standard deviation alpha)  = 5%, then *Z*_1−*α*_ = 1.64); *Z*_1−*β*_ is the value in table *Z* (if *β* (standard deviation beta) = 20%, then *Z*_1−*β*_ = 0.84); ln is the natural logarithm; and *ρ*_0_is the alleged correlation coefficient of the studied variable = 0.25.

### 2.5. Data Collection

Medical records of subjects with normal and low birth weight who were born at Prof. Dr. R. D. Kandou General Hospital Manado from 2010 to 2013 and resided in the city of Manado were reviewed to obtain the required data for analyses. The parents were contacted and informed about the purpose and the benefits of their participation in this study. Once the parents provided the consent, an experienced physician visited their home to perform a physical examination, anthropometric assessment, and blood pressure measurement and was blinded to the subjects' birth weight data. The children who met the inclusion criteria were enrolled in the cohort and were subsequently evaluated for the hs-CRP level. Blood samples were drawn by experienced laboratory staff. Blood sample collection was performed with sterile instruments and started by disinfecting the area for needle insertion using 70% alcohol swab, followed by taking a total of 3 ml venous blood samples drawn from the medial cubital vein using a syringe connected to a vacutainer. The special blood sample tube was labeled with the patient's identity. All the regarded data obtained through a series of interview, physical examination, anthropometric assessment, and hs-CRP measurement were subsequently analyzed.

### 2.6. Data Management and Statistical Analyses

Descriptive analysis was conducted in all study variables that were classified into three main birth weight categories: low birth weight appropriate for gestational age (LBW AGA), low birth weight small for gestational age (LBW SGA), and normal birth weight appropriate for gestational age (NBW AGA). LBW AGA is defined as babies with birth weight <2500 grams and gestational age at the 10th and 90th percentile of the Lubchenko curve. LBW SGA is defined as babies with birth weight <2500 grams and gestational age below the 10th percentile according to the Lubchenko curve. NBW AGA is defined as babies with birth weight between 2500–4000 grams and gestational age at the 10–90th percentile of the Lubchenko curve. Comparison among the three birth weight groups with blood pressure and hs-CRP level was done using the ANOVA test. Independent *t*-test was employed for assessing the association between low birth weight and normal birth weight groups with both hs-CRP level and blood pressure. Simple linear regression and multivariate regression analysis was performed to investigate the correlation between birth weight with both blood pressure and hs-CRP level. All the statistical analyses were performed using SPSS software version 25.0.

## 3. Results

The study was conducted from April 2019 to September 2019 in children aged 9–12 years who were born around 2007–2010 at Prof. Dr. R. D. Kandou General Hospital and resided in Manado. A total of 120 children who met the inclusion criteria were enrolled in this study. All of the eligible children were further grouped based on their birth weight which resulted in the following categorization: 40 children with low birth weight small for gestational age, 40 children with low birth weight appropriate for gestational age, and 40 children with normal birth weight. Baseline characteristics of this study are shown in Table 1.

Hs-CRP levels can be classified as low risk of chronic subtle inflammation if hs-CRP levels are <1 mg/L, moderate risk of chronic subtle inflammation if hs-CRP levels are 1–3 mg/L, and high risk of chronic subtle inflammation if hs-CRP levels are >3 mg/L. Serum hs-CRP evaluation showed that out of 40 children with a history of LBW SGA, 19 children (47.5%) were considered having mild risk, 17 children (42.5%) with moderate risk, and 4 children (10%) with high risk. On the other hand, serum hs-CRP analysis in 40 children with a history of LBW AGA revealed 20 children (50%) with mild, 17 children (42.5%) with moderate, and 3 children (7.5%) with high risk. The last group showed all the 40 children (100%) with low risk regarding their serum hs-CRP level.

**Figure 1:**
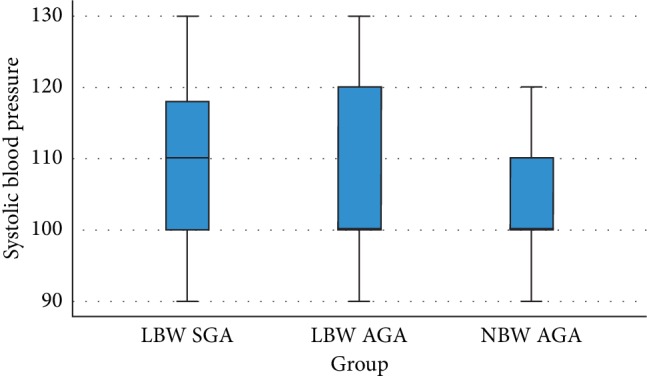
Box plot association between birth weight and systolic blood pressure of children with a history of LBW AGA, LBW SGA, and NBW AGA.

**Figure 2:**
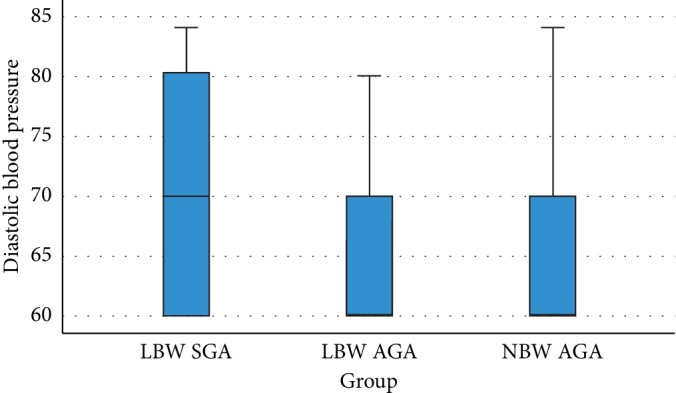
Box plot association between birth weight and diastolic blood pressure of children with a history of LBW AGA, LBW SGA, and NBW AGA.

Table 2 presents the data indicating that there is no significant association between each study group and both systolic (*p*=0.76) and diastolic (*p*=0.109) blood pressure. The graphic presentations of these results are shown in Figures 1 and 2.

The results from the analysis for the association between low and normal birth weight with systolic blood pressure using independent *t*-test have statistical significance with *p*=0.007. Meanwhile, no significant results were found for the association with diastolic blood pressure (*p*=0.254) as shown in Table 3.

**Figure 3:**
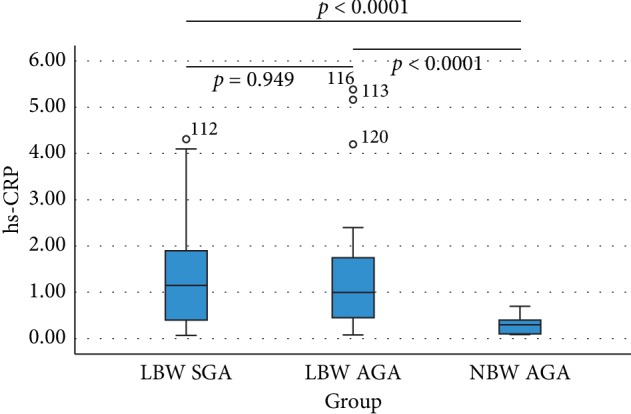
Box plot association between birth weight and hs-CRP level in children with history of LBW SGA, LBW AGA, and NBW AGA.

The results from the analysis regarding the association between children with LBW SGA, LBW AGA, NBW AGA, and serum hs-CRP level using ANOVA test showed the *p* value of <0.0001 (Table 4). The graphic illustration for the association between the three variables is depicted in Figure 3. There is no significant difference between serum hs-CRP level with the LBW AGA and LBW SGA group (*p*=0.949). There is a significant difference between serum hs-CRP level in the LBW SGA and NBW AGA group (*p* < 0.0001). There is a significant difference between serum hs-CRP level in the LBW AGA and NBW AGA group (*p* < 0.0001).

Independent *t*-test analysis for the difference between the low birth weight group and the normal birth weight group counterpart with serum hs-CRP level resulted in a significant result with the *p* value of <0.0001 (Table 5).

### 3.1. Association between Birth Weight and hs-CRP Level

**Figure 4:**
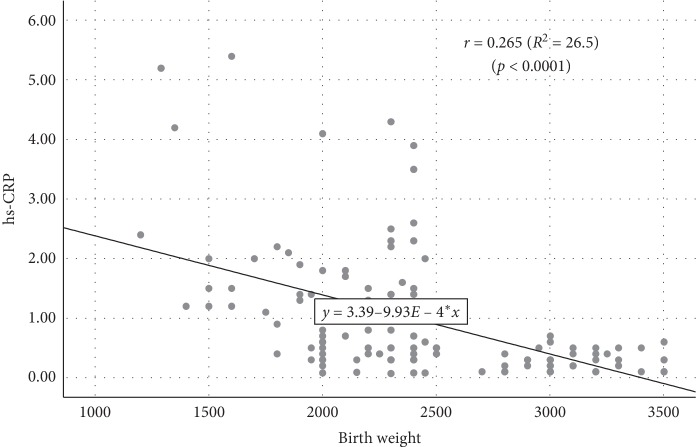
Scatterplot diagram for the association between birth weight and serum hs-CRP level.

Simple linear regression analysis indicated a strongly significant influence of birth weight (BW) on the serum hs-CRP level. The result showed that the heavier the birth weight, the lower the serum hs-CRP level (Figure 4). Every 1 g increase in birth weight results in a decrease of serum hs-CRP level of 0.001 mg/L (birth weight = 3.38–0.001 hs-CRP, *r* = 0.265, *p* < 0.0001).

### 3.2. Association between Birth Weight and Systolic (SBP) and Diastolic (DBP) Blood Pressure

**Figure 5:**
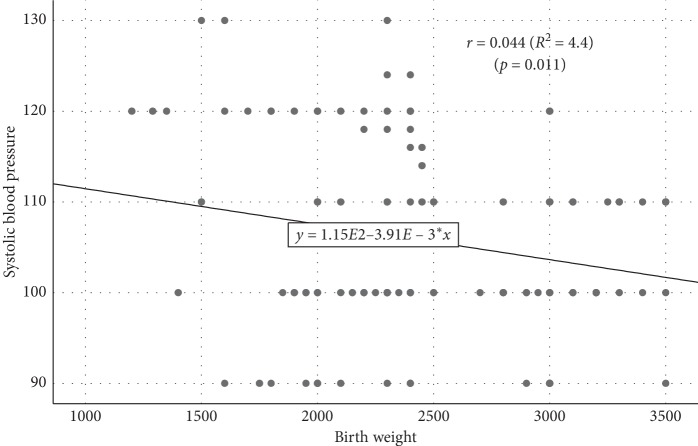
Scatterplot diagram for the association between birth weight and systolic blood pressure.

Simple linear regression analysis demonstrated a significant influence of BW on SBP as shown in Figure 5. The larger the BW, the lower the SBP. Every 1 g increase in BW is attributed to 0.004 mmHg decrease in SBP (birth weight = 115.14–0.004 SBP, *r* = 0.044, *p*=0.011).

**Figure 6:**
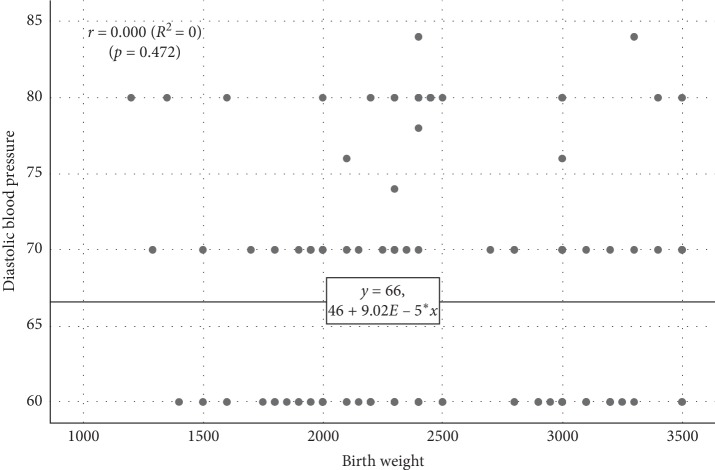
Scatterplot diagram for the association between birth weight and diastolic blood pressure.

On the contrary, the linear regression analysis showed no significant influence of BW on the DBP (Figure 6) (birth weight = 66.46 + 0.00009 DBP, *r* = 0.000, *p*=0.472).

### 3.3. Association between Serum hs-CRP Level and Systolic and Diastolic Blood Pressure

**Figure 7:**
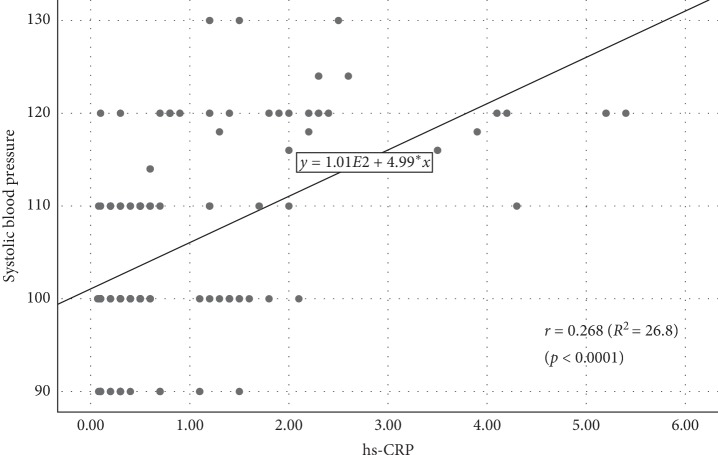
Scatterplot diagram for the association between hs-CRP level and systolic blood pressure.

Simple linear regression analysis showed a significant positive correlation between hs-CRP and SBP (Figure 7). The higher the serum hs-CRP level, the higher the SBP. An increase in hs-CRP by 1 mg/L increases the SBP by 4.99 mmHg (hs-CRP = 101.07 + 4.99 SBP, *r* = 0.268, *p* < 0.0001).

**Figure 8:**
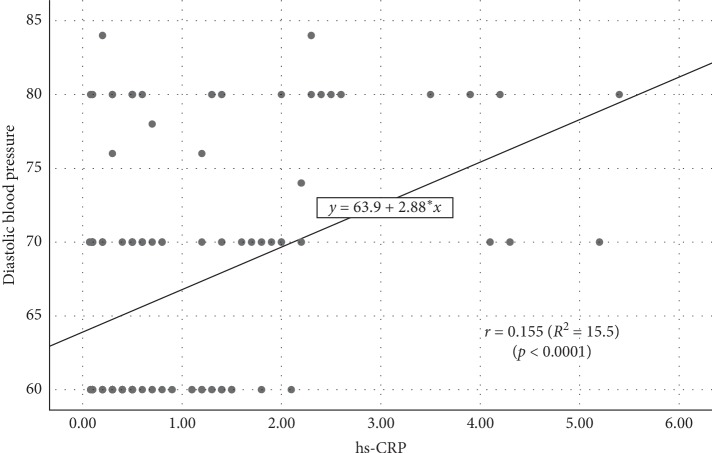
Scatterplot diagram for the association between hs-CRP level and diastolic blood pressure.

Simple linear regression analysis resulted in a significant positive correlation between hs-CRP and DBP (Figure 8). The higher the serum hs-CRP level, the higher the DBP. An increase in hs-CRP by 1 mg/L increases the DBP by 2.88 mmHg (hs-CRP = 63.90 + 2.88 DBP, *r* = 0.155, *p* < 0.0001).

The multivariate regression analysis of hs-CRP and birth weight with systolic and diastolic blood pressure with control variables such as child's age, sex, weight, and height is presented in Tables 6 and 7.

The results of multivariate regression analysis showed that only the hs-CRP variable is associated with systolic blood pressure in children (*p* < 0.0001), whereas the diastolic blood pressure is influenced by birth weight and hs-CRP variables (*p*=0.004 and *p* < 0.0001, respectively).

## 4. Discussion

The association between birth weight and age-stratified systolic blood pressure shows fetal programming as the underlying mechanism which includes permanent alteration in the vascular structure, abnormality in nephrogenesis, and effects of glucocorticoid hormones, as well as increased sympathetic nervous system activities [9].

The growth pattern associated with the development of hypertension is characterized by the slow intrauterine growth followed by accelerating “compensation” mechanism to increase body weight and height. The other mechanisms that contribute to the development of hypertension involve arterial compliance and elastogenesis. Impaired arterial compliance is discovered among patients with low birth weight owing to the reduction in elastin content constructing the vascular wall since their intrauterine growth. Fetal exposure to maternal glucocorticoid is associated with hypertension in adulthood. The mechanism underlying the effects of glucocorticoid exposure on hypertension that occurred later in life is still a subject of research. Increased glucocorticoid sensitivity, however, could trigger an increase in angiotensin-converting enzyme activity that results in increased angiotensin-II level that plays a major role in maintaining blood pressure homeostasis [10].

Statistical analysis for the association between each birth weight group (LBW SGA, LBW AGA, and NBW AGA) and both systolic (*p*=0.76) and diastolic (*p*=0.109) blood pressure showed no significant association. On the contrary, the analysis regarding the relationship between low and normal birth weight children with the systolic blood pressure succeeded in reaching statistical significance with the *p* value of 0.007. This result indicates a significant difference between the two birth weight groups in terms of systolic blood pressure. As for the diastolic blood pressure, statistical analysis showed a nonsignificant association (*p*=0.254).

Recent evidence proves that low birth weight reflects a poor intrauterine environment, which is associated with the disappearance of nephron endowment and other pathophysiologic mechanisms predisposing to elevated blood pressure. The average number of the nephron in normal individuals ranges from 300,000 to 1.1 million, and its normal development reaches 60% by the end of the last trimester [11]. *β*-Adrenergic receptors (*β*-AR) play an important role in the regulation of various organ systems, including cardiovascular, pulmonary, endocrine, and sympathetic nervous systems. Polymorphism of the *β*-AR gene is attributed to several variables associated with increased risk of cardiovascular events, including hemodynamic parameters such as heart rate, vasodilation, and blood pressure. Moreover, the *β*-AR gene is attributed to preterm birth in children [12].

Formation and development of a perfect kidney in the fetus are achieved at 36 weeks gestational age, and no more nephrons will be formed afterward. Intrauterine nephron formation is strongly influenced by intrauterine fetal growth that is linked with maternal nutritional status, vitamin intake, intrauterine infection, exposure to antibiotics, and smoking and alcohol intake during pregnancy [13]. Brenner et al. [14, 15] reported that impairment in intrauterine renal growth and development resulted in a decreased number of healthy and functioning nephrons which contribute to the development of hypertension later in life. This concept is further known as Brenner hypothesis or nephron-underdosing theory. Low birth weight possesses a positive correlation with renal impairment and decreased the nephron number at birth [13].

In a study conducted by Ediriweera et al., in 2017, LBW showed a significant effect on hypertension, particularly its correlation with elevated systolic blood pressure in adulthood, but not directly with high diastolic blood pressure [16]. Stock et al. in their study conducted in 930 Austrian adolescents with a history of premature birth or small for gestational age showed that their SBP and DBP are significantly higher than children at their age with NBW AGA [17]. A cross-sectional study performed by Rahayu et al. in North Sumatera concluded that birth weight has a negative association with SBP and DBP [18]. Huang and colleagues in Taiwan found that pure preterm and preterm with small for gestational age groups are at a significantly higher risk of developing hypertension compared to the small for gestational age group. This phenomenon can be explained by the impaired glomerular formation process in preterm babies [19]. The findings from Ting Huang's study support the results of a meta-analysis by de Jong et al. who found a significant association between prematurity and increased systolic blood pressure among teenagers aged 18 years [20].

Linear regression analysis showed a significant influence of BW on SBP. The higher the BW, the lower the SBP. Every 1 g increase in BW correlates with a reduction in SBP by 0.004 mmHg. In the Bogalusa Heart study, birth weight negatively correlated with blood pressure level in adulthood and long-term blood pressure level [16]. This finding is also supported by Chen W et al. in 2012 who found that LBW was significantly associated with increased blood pressure during adulthood [21]. In a study involving 9921 children in England, there was a significant inverse association between systolic blood pressure and birth weight. Data analysis performed by Seidmann et al. resulted in a weak association between birth weight and blood pressure. Whincup and colleagues studied the blood pressure level in 9–11-year-old children and its relation to their birth weight and found that the regression coefficient for birth weight was as high as 2.80 mmHg/kg birth weight. A similar study performed by Taylor et al. reported the regression coefficient of 1.48 mmHg/kg birth weight. Huxley et al. conducted a review on all published studies and concluded that an increase in birth weight by as much as 1 kg correlates with 1-2 mmHg reduction in systolic blood pressure [22].

The result from the present study indicates a significant positive correlation between birth weight status and hs-CRP level during childhood. Similar results were also discovered for both low birth weight and normal birth weight groups (*p* < 0.0001). Our results support the results of the previous study by Turoni et al. which reported that serum hs-CRP level is higher among children with a history of low birth weight compared to the control group [23].

The results of this study showed no significant difference in serum hs-CRP level between children with LBW SGA and children with LBW AGA (*p*=0.949). There was a significant difference in hs-CRP level between children with LBW SGA and children with NBW AGA (*p* < 0.0001). There was also a significant difference in hs-CRP level between the LBW AGA and NBW AGA groups (*p* < 0.0001). Turoni et al. also reported no significant difference in hs-CRP level between the LBW SGA and LBW AGA groups. The difference was only found when the serum hs-CRP level was compared with the control group. Hs-CRP is not only considered a marker for cardiovascular risk, but its increase also reflects a cardiovascular damage [23].

Regression analysis resulted in a highly significant positive correlation between hs-CRP and SBP and DBP. The higher the serum hs-CRP level, the higher the SBP and DBP. An increase in hs-CRP by 1 mg/L increases the SBP by 4.99 mmHg and DBP by 2.88 mmHg. These results support the previous discovery by Wasilewska et al. in Poland that involved children aged 10–19 years with prehypertension and hypertension where the hs-CRP level among these groups were higher than the control group (*p* < 0.01), and the level was higher in the hypertension group compared to the prehypertension group (*p* < 0.05) [21]. Lande et al. in New York performed a cross-sectional study in children and adolescents aged 8–17 years and found that children with serum CRP level of ≥3 mg/L had a higher SBP compared to those with serum CRP level of ≤3 mg/L, while DBP did not significantly differ between the two groups [24]. A study by Rondo et al., in Brazil, was conducted in a total of 459 children aged 5–8 years and resulted in a significant positive correlation between hs-CRP level and systolic blood pressure (*p*=0.03) [25].

The mechanisms on how CRP affects the vascular damage would be best attributed to angiogenesis, endothelial damage, oxidative stress, and vascular remodeling. Inflammation is considered a key mechanism in the pathogenesis of atherosclerosis and inflammatory process and also significantly associated with the development of arterial hypertension, heart failure, and valvular heart disease, as well as atrial fibrillation. The inflammatory cells increase the metabolic activities around the vascular wall making a more acidic medium that accelerates cellular apoptosis. By activating the angiotensin-aldosterone system, angiotensin-I, and angiotensin-II receptors, CRP induces angiotensin-mediated proatherogenic activities, directly and indirectly stimulates structural and functional modification of the arterial wall, vascular and cardiac remodeling, vascular stiffening, an increase in peripheral vascular resistance, and impairs regulatory mechanism of arterial blood pressure. CRP induces matrix metalloproteinase (MMP) activities (leading to the destruction of collagen) in macrophages and endothelial cells, as well as suppressing the MMP inhibitors within the tissue [26].

Preterm babies live outside the uterine cavity during the normal fetal developmental period from their birth until reaching the adequate gestational age (40 weeks after menstrual age) and usually spend several weeks to months after birth in a neonatal intensive care unit. Poor perinatal condition also affects BP through fetal programming mechanism [20] Shah et al. in India linked brain injury during the perinatal period with increased blood pressure at the later age [27].

Several limitations of this study include blood pressure measurement was only carried out once at a time (morning BP) so that the possibility of white coat effect might have caused bias, whereas several studies recommend performing an ambulatory blood pressure monitoring (24 hours). The serum hs-CRP level was measured only once. In adults, the AHA and CDC recommend performing CRP level for at least twice with a minimum of a 2-week interval to determine the cardiovascular risk. This study did not include both maternal and environmental factors in the analyses that might also contribute to increasing the risk of having increased blood pressure in children.

## 5. Conclusion

Children with a history of low birth weight are significantly associated with higher systolic blood pressure during childhood. Low birth weight also significantly correlates with serum hs-CRP level. The higher the birth weight, the lower the hs-CRP level. An increase in hs-CRP by 1 mg/L increases the SBP by 4.99 mmHg and DBP by 2.88 mmHg. A comprehensive education must be given to the families who have children with low birth weight to reduce the risk of developing hypertension later in their life.

## Figures and Tables

**Table 1:** Baseline characteristics of the study participants.

	LBW SGA (*n* = 40)	LBW AGA (*n* = 40)	NBW AGA (*n* = 40)
Gender			
Male	16 (40%)	23 (57.5%)	25 (62.5%)
Female	24 (60%)	17 (42.5%)	15 (37.5%)
Mean age (years)	11.05 ± 1.34	11.10 ± 1.75	11.13 ± 1.32
Mean weight (kg)	29.63 ± 7.25	31.11 ± 8.52	32.45 ± 7.57
Mean height (cm)	134.50 ± 10.62	136.25 ± 12.64	138.38 ± 10.41
Mean systolic blood pressure (mmHg)	108.20 ± 10.55	106.50 ± 12.31	103 ± 7.57
Mean diastolic blood pressure (mmHg)	68.80 ± 9.13	65.25 ± 6.79	66 ± 7.49
hs-CRP level (mg/L)	1.31 ± 1.14	1.30 ± 1.23	0.28 ± 0.16

**Table 2:** Association between children with LBW SGA, LBW AGA, and NBW AGA with systolic and diastolic blood pressure.

Blood pressure	LBW AGA (*n* = 40)	LBW SGA (*n* = 40)	NBW AGA (*n* = 40)	*p* value
Systolic blood pressure (mmHg, mean ± standard deviation)	106.50 ± 12.31	108.20 ± 10.55	103.00 ± 7.58	0.76^*∗*^
Diastolic blood pressure (mmHg, mean ± standard deviation)	65.25 ± 6.79	68.80 ± 9.14	66.00 ± 7.50	0.109^*∗*^

^*∗*^ANOVA.

**Table 3:** Association between children with low and normal birth weight with systolic and diastolic blood pressure.

Blood pressure	Low birth weight (*n* = 80)	Normal birth weight (*n* = 40)	*p* value
Systolic blood pressure (mmHg, mean ± standard deviation)	107.35 ± 11.42	103.0 ± 7.58	0.007^*∗*^
Diastolic blood pressure (mmHg, mean ± standard deviation)	67.02 ± 8.19	66.0 ± 7.50	0.254^*∗*^

^*∗*^Independent Student's *t*-test.

**Table 4:** Association between the three birth weight groups and serum hs-CRP level.

hs-CRP level	LBW AGA (*n* = 40)	LBW SGA (*n* = 40)	NBW AGA (*n* = 40)	*p* value
hs-CRP (mg/L, mean ± standard deviation)	1.30 ± 1.24	1.32 ± 1.15	0.285 ± 0.17	<0.0001^*∗*^

^*∗*^ANOVA.

**Table 5:** The difference in serum hs-CRP level between the low birth weight and normal birth weight group.

hs-CRP level	Low birth weight (*n* = 80)	Normal birth weight (*n* = 40)	*p* value
hs-CRP (mg/L, mean ± standard deviation)	1.31 ± 1.19	0.29 ± 0.17	<0.0001^*∗*^

^*∗*^Independent Student's *t*-test.

**Table 6:** Relationship of hs-CRP and birth weight with systolic blood pressure with control variables such as child's age, sex, weight, and height.

Variable	Coefficient B	Standard error	Coefficient beta	*t*	*p*
Constant	84.515	24.267		3.483	0.001
Birth weight	0.001	0.002	0.066	0.772	0.442
hs-CRP	5.977	0.843	0.620	7.087	0.0001
Age	0.000	0.003	0.009	0.126	0.900
Weight	0.479	0.365	0.357	1.310	0.193
Height	−0.012	0.253	−0.013	−0.046	0.963
Sex	−1.360	1.563	−0.065	−0.870	0.386

**Table 7:** Relationship of hs-CRP and birth weight with diastolic blood pressure with control variables such as child's age, sex, weight, and height.

Variable	Coefficient B	Standard error	Coefficient beta	*t*	*p*
Constant	70.275	19.901		3.531	0.001
Birth weight	0.004	0.001	0.274	2.959	0.004
hs-CRP	4.338	0.692	0.593	6.272	0.0001
Age	−0.001	0.002	−0.037	−0.462	0.645
Weight	0.587	0.300	0.577	1.958	0.053
Height	−0.251	0.207	−0.357	−1.213	0.228
Sex	−1.830	1.281	−0.115	−1.428	0.156

## Data Availability

The datasets used and/or analysed during the current study are available from the corresponding author on reasonable request.

## References

[1] Sutherland M. R., Bertagnolli M., Lukaszewski M.-A. (2014). Preterm birth and hypertension risk the oxidative stress paradigm. *Hypertension*.

[2] Global W. HO. (2014). *Nutrition Targets 2025: Low Birth Weight Policy Brief*.

[3] Kramer M. S., Bhatia J., Bhutta Z. A., Kalhan S. C. (2013). The epidemiology of low birthweight. *Maternal and Child Nutrition: The First 1000 Days*.

[4] Hartanto S., Mustadjab I. Profil bayi berat lahir rendah di ruang perinatologi RSUP manado.

[5] Barker D. J. P. (2004). Developmental origins of adult disease. *Journal of the American College of Nutrition*.

[6] Hughson M., Farris A. B., Douglas-Denton R., Hoy W. E., Bertram J. F. (2003). Glomerular number and size in autopsy kidneys: the relationship to birth weight. *Kidney International*.

[7] Parinello C. M., Lutsey P. L., Ballantyne C. M., Folsom A. R., Pankow J. S., Selvin E. (2015). Six-year change in high sensitivity C-reactive protein and risk of diabetes, cardiovascular disease, and mortality. *American Heart Journal*.

[8] Rosner B. (2016). *Fundamentals of Biostatistics*.

[9] Chen W., Srinivasan S. R., Berenson G. S. (2010). Amplification of the association between birthweight and blood pressure with age: the Bogalusa heart study. *Journal of Hypertension*.

[10] Lackland D. T., Egan B. M., Ferguson P. L. (2003). Low birth weight as a risk factor for hypertension. *The Journal of Clinical Hypertension*.

[11] Salmi I. A., Shaheen F. A. M., Hannawi S. (2019). Birth weight, gestational age, and blood pressure: early life management strategy and population health perspective. *Saudi Journal of Kidney Diseases and Transplantation*.

[12] Chen W., Srinivasan S. R., Hallman D. M., Berenson G. S. (2010). The relationship between birthweight and longitudinal changes of blood pressure is modulated by beta-adrenergic receptor genes: the Bogalusa heart study. *Journal of Biomedicine and Biotechnology*.

[13] Spencer J., Wang Z., Hoy W. (2001). Low birth weight and reduced renal volume in aboriginal children. *American Journal of Kidney Diseases*.

[14] Brenner B. M., Milford E. L. (1993). Nephron underdosing: a programmed cause of chronic renal allograft failure. *American Journal of Kidney Diseases*.

[15] Reyes L., Manalich R. (2005). Long term consequences of low birth weight. *Kidney International*.

[16] Ediriweera D. S., Dilina N., Perera U., Flores F., Samita S. (2017). Risk of low birth weight on adulthood hypertension—evidence from a tertiary care hospital in a South Asian country, Sri Lanka: a retrospective cohort study. *BMC Public Health*.

[17] Stock K., Schmid A., Griesmaier E. (2018). The impact of being born preterm or small for gestational age on early vascular aging in adolescents. *The Journal of Pediatrics*.

[18] Rahayu S., Rusdidjas R., Ramayati R., Ramayani O. R., Siregar R. (2015). Relationship between childhood blood pressure and birth weight. *Paediatrica Indonesiana*.

[19] Huang Y.-T., Lin H.-Y., Wang C.-H., Su B.-H., Lin C.-C. (2018). Association of preterm birth and small for gestational age with metabolic outcomes in children and adolescents: a population-based cohort study from Taiwan. *Pediatrics & Neonatology*.

[20] de Jong F., Monuteaux M. C., van Elburg R. M., Gillman M. W., Belfort M. B. (2012). Systematic review and meta-analysis of preterm birth and later systolic blood pressure. *Hypertension*.

[21] Wasilewska A., Tenderenda E., Taranta-Janusz K., Zoch-Zwierz W. (2010). High sensitivity C-reactive protein and mean platelet volume in paediatric hypertension. *Pediatric Nephrology*.

[22] Falkner B. (2002). Birth weight as a predictor of future hypertension. *American Journal of Hypertension*.

[23] Turoni C. J., Chaila Z., Chahla R., Bazan de Casella M. C., Peral de Bruno M. (2016). Vascular function in children with low birthweight and its relationship with early markers of cardiovascular risk. *Hormone Research in Paediatrics*.

[24] Lande M. B., Pearson T. A., Vermilion R. P., Auinger P., Fernandez I. D. (2008). Elevated blood pressure, race/ethnicity, and C-reactive protein levels in children and adolescents. *Pediatrics*.

[25] Rondó P. H., Pereira J. A., Lemos J. O. (2013). High sensitivity C-reactive protein concentrations, birthweight and cardiovascular risk markers in Brazilian children. *European Journal of Clinical Nutrition*.

[26] Adakauskine D., Ciginskiene A., Adakauskaite A., Pentiokiniene D., Slapikas R., Ceponiene I. (2016). Clinical relevance of high sensitivity C-reactive protein in cardiology. *Medicina*.

[27] Shah A. B., Hashmi S. S., Sahulee R., Pannu H., Gupta-Malhotra M. (2015). Characteristics of systemic hypertension in preterm children. *The Journal of Clinical Hypertension*.

